# 

**Published:** 1889-10-19

**Authors:** 


					Ti
k
?J?'9E ONE penny.
??
Vo), ?n.-
?October 19th, 1889.
*ratt?mis?i * . General Post Office as a Newspaper for
n in the United Kingdom and Abroad.
Per Annum, 6s. 6d., post free. (J
Monthly Parts, 6d.; post free' 7?d.
Ditto per Annum, 7s. 6d., post free.

				

